# Valosin-Containing Protein (VCP/p97)-Expression Correlates with Prognosis of HPV- Negative Oropharyngeal Squamous Cell Carcinoma (OSCC)

**DOI:** 10.1371/journal.pone.0114170

**Published:** 2014-12-02

**Authors:** Moritz F. Meyer, Inga M. C. Seuthe, Uta Drebber, Oliver Siefer, Matthias Kreppel, Marcus O. Klein, Stefanie Mikolajczak, Jens Peter Klussmann, Simon F. Preuss, Christian U. Huebbers

**Affiliations:** 1 Department of Otorhinolaryngology, Head and Neck Surgery, University of Cologne, Cologne, Germany; 2 Department of Pathology, University of Cologne, Cologne, Germany; 3 Jean-Uhmacher Institute, University of Cologne, Cologne, Germany; 4 Department for Oral and Maxillofacial Plastic Surgery, University of Cologne, Cologne, Germany; 5 Clinic for Oral and Maxillofacial Plastic Surgery, Dusseldorf, Germany; 6 Department of Otorhinolaryngology, Head and Neck Surgery, University of Giessen, Giessen, Germany; Duke Cancer Institute, United States of America

## Abstract

Valosin-containing protein (VCP)/p97 has been shown to be associated with antiapoptotic function via activation of the nuclear factor-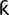
B (NF
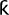
B) signaling pathway and with metastasizing of tumors in several studies. VCP is located on chromosome 9p13-p12, a region often deleted in oropharyngeal squamous cell carcinoma (OSCC). The clinical significance of VCP expression in OSCC however remains unclear. In this study, expression of VCP was determined in 106 patients (77 male (71.3%) and 31 female (28.7%); age-range: 34–79 years (mean age 57 years)) by immunohistochemistry and in a subset of 15 patients by quantitative PCR. HPV-DNA was detected by polymerase chain reaction and p16^INK4a^ immunohistochemistry. The experimental findings were correlated with clinico-pathological data and survival parameters. 47.2% of all OSCC specimens were analyzed as negative or weak staining intensity for VCP. 52.8% of all specimens showed a high staining intensity for VCP. 73.1% of all patients were tested HPV-negative, 26.9% were HPV-positive. The 5-year disease-free and overall survival probabilities of all patients were 71.2% and 55.7%, respectively. No correlation could be found between HPV-status and VCP expression. VCP overexpression in HPV-negative patients was associated with significantly better 5-year disease-free survival (86.4% vs., 45.6%, p = 0.017). The level of VCP-intensity determined by immunohistochemistry could be an additional prognostic marker in HPV-negative OSCC. VCP expression seems not to correlate with the HPV-status.

## Introduction

Oropharyngeal squamous cell carcinoma (OSCC) is the most frequent malignancy of the head and neck region [1]. The incidence of OSCC is still rising [2], [3]. Genetic features and environmental factors may contribute to the incidence of OSCC. Beside the established risk factors tobacco and alcohol consumption [4]–[6] there are several studies that suggest that a subset of OSCC are associated with oncogenic human papillomavirus (HPV) infection, in particular high-risk HPV type 16 [7]–[9]. Therefore, current studies distinguish a group of HPV-positive and a group of HPV-negative carcinomas [7]–[9]. As a result of a better response to radiotherapy and chemotherapy in HPV-induced OSCC, recent studies show that the prognosis is superior in HPV-positive carcinomas compared to HPV-negative carcinomas [4]. However, the 5-year overall survival rate remains poor in these patients [10] and ranges from 45% to 58% [11]. The tumor-node-metastasis (TNM) staging system is a worldwide accepted classification system and is based on primary tumor classification (T), the number, size and laterality of lymph node metastasis (N) and on the presence of distant metastasis (M). Treatment guidelines for OSCC have been suggested based on clinical (e.g. TNM) and histological characteristics (e.g. Grading). However, these parameters are not sufficient as precise prognostic indicators. Thus, several clinical and molecular factors in carcinogenesis and prognosis have been proposed as prognostic biomarkers in oropharyngeal cancer [12]–[15].

Valosin-containing protein (VCP, also known as p97) has been shown to be associated with metastatic potential of cancer cells using the mRNA subtraction technique [16]. As VCP is member of the ATPases associated with various cellular activities (AAA) superfamily, it is suggested that VCP is involved in the ubiquitindependent proteasome degredation pathway of 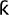
Ba (I
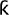
Ba), an inhibitor of nuclear factor-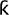
B (NF
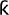
B) [17]–[19]. An up-regulation of cell-proliferation and down-regulation of cell death in human cancer cells is effected [18]. These findings suggest that the expression of VCP has the potential to predict metastasis and prognosis in various human cancers [19]. It has been reported as a suitable biomarker to predict prognosis in esophageal, gastric, prostate and lung carcinoma [18]–[21]. Since VCP was shown to be regulated by the E6-E6AP-PTPN3 network, analysis of VCP expression levels in HPV-positive OSCC specimens might be of particular interest [22]. On the other hand, VCP is located on chromosome 9p13-p12, a region that is often deleted in HPV-negative OSCC and is associated with a superior disease free and overall survival [23].

The aim of this study was to analyze VCP-expression using immunohistochemistry staining in HPV-negative and HPV-positive OSCC specimens and to correlate VCP expression levels with clinico-pathological features and survival of these patients.

## Materials and Methods

### Ethics Statement

Patient material was used according to the code for proper secondary use of human tissue. The study was approved by the local ethics committee of the University of Cologne medical faculty and was according to the latest version of the Declaration of Helsinki. Written, informed consent had been obtained from all patients.

### Patients

Tissue samples from 106 patients were obtained during curative resection for primary OSCC between 2000 and 2005 at the Department of Otorhinolaryngology, Head and Neck Surgery, University of Cologne. There were 75 (70.8%) males and 31 (29.2%) females. The age ranged from 34 to 79 years (mean age 57 years). The clinical and pathological staging of the OSCC was determined via TNM and the seventh edition of the American Joint Committee on cancer staging [24]. The pathological staging and grading was assessed by the pathologists of the Department of pathology, University of Cologne. All patients underwent regular follow-up examinations at the oncological outpatient clinic in intervals of 2–4 months.

### Tissue samples

Additional samples obtained from the OSCC lesions were fixed in 4% formalin and processed routinely for paraffin embedding [18], [19]. Histological sections were cut at 6 µm and stained with hematoxylin and eosin (H and E) and immunoperoxidase procedures (avidin-biotin complex method). Inclusion criteria of the samples were sufficient-embedded tumor tissue (over 70%).

### Immunohistochemistry

For staining of VCP, 6 µm sections were cut by a microtome and placed on Superfrost Plus objective plates (Carl Roth, Karlsruhe, Germany). These sections were dried in an incubator at 37°C for 12 h. In the next step the sections were separated from paraffin. This was done by washing the sections with Roti-Histol two times for 5 min (Carls Roth, Karlsruhe, Germany). Afterwards the sections were washed two times with Isopropanol, alcohol in descending concentrations (100%, 96%, 80%, 70% and 40% ethanol) and aqua destillata for another 5 min, respectively (Rehydration). After rehydration, the sections were incubated for 12 h in 0.05 M TBS pH 7.4 in an incubator at 70°C.

**Figure 1:**
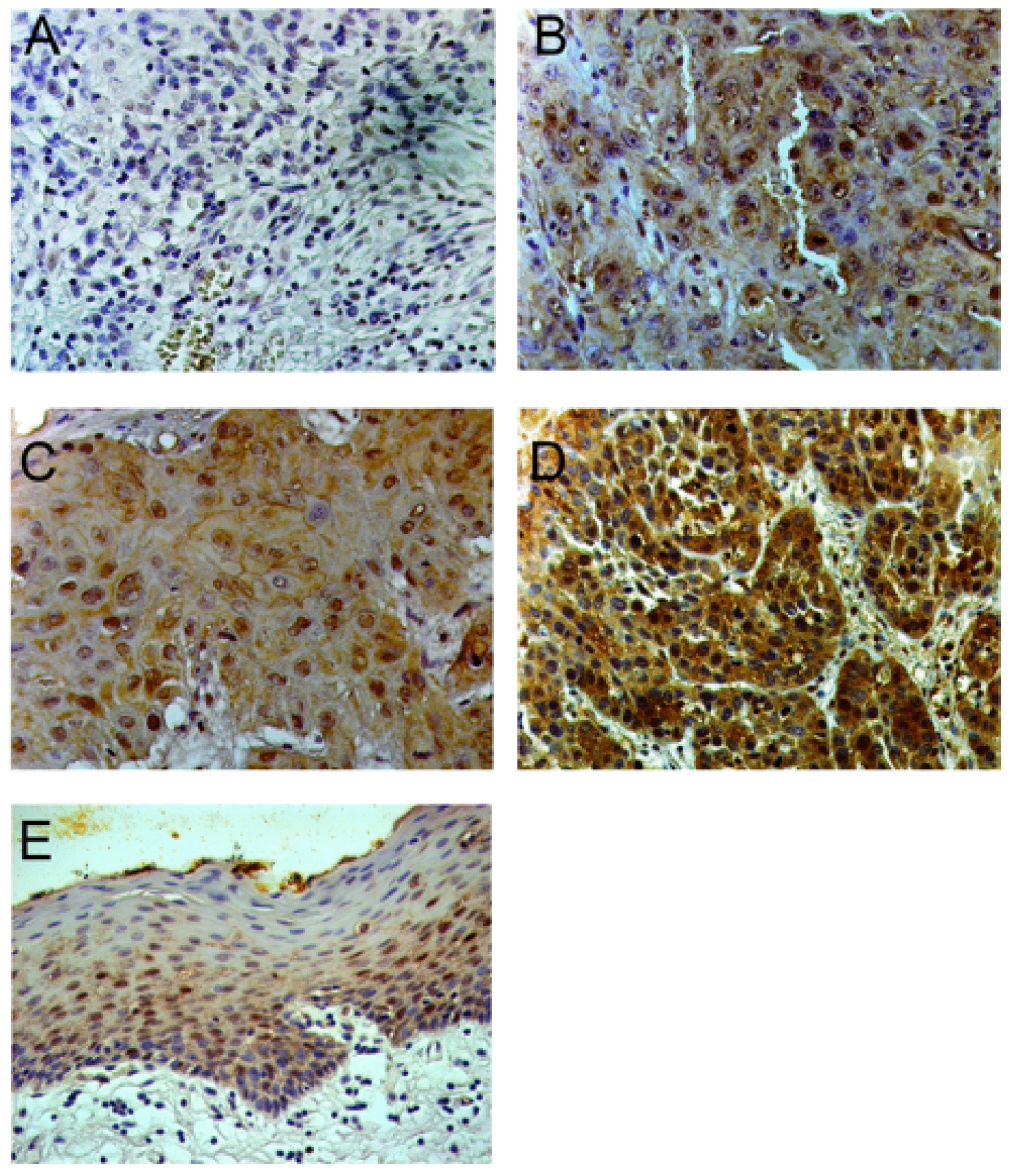
VCP-expression level in OSCC patients (original magnification, x400). **A:** expression level 0, **B:** expression level 1, **C:** expression level 2, **D:** expression level 3. In statistical analysis level 0 and 1 were summarized to level 0–1 and level 2 and 3 were summarized to level 2–3. **E:** VCP expression in non-carcinoma endothelial cells.

Before staining the sections were washed in a phosphate-buffered saline (PBS) for 5 min and in a solution of 3% H_2_O_2_ in methanol to intend a blocking of the peroxidase. Mouse monoclonal Anti-VCP (p97) antibody (BD Transduction Laboratories, Heidelberg, Germany) was used as the primary antibody at a dilution of 1∶500 in PBS. For negative control sections were incubated with hourse anti-mouse mouse antibody at a dilution of 1∶250 (BioLogo, Kronshagen, Germany) in PBS. Primary antibodies were detected by the avidin-biotinylated peroxidase complex (ABC) procedure (Vectastain-Elite-ABC kit; Vector Laboratories, Burlingame, USA) and peroxidase activity was visualized using diaminobenzidine/H_2_O_2_ (BD Biosciences, Heidelberg, Germany). Sections were counterstained with hematoxylin and mounted in Histofluid (Marienfeld, Lauda-Koenigshofen, Germany). Each analysis included negative controls (no primary or no secondary antibody, respectively). Staining of normal epithelium served as positive control. Stained areas were analyzed in a blind manner without any prior information of clinical findings of the patients (CUH, IMCS and SFP) and consensus was achieved. We analyzed the absolute intensity of counterstaining without comparing to non-carcinoma endothelial cells and classified them as level 0 (without counterstaining), level 1 (low grade), level 2 (moderate grade) and level 3 (high grade) (Figure 1). In statistical analysis level 0 and 1 were summarized to level 0–1 and level 2 and 3 were summarized to level 2–3.

### P16 immunohistochemistry

Immunohistochemistry of the surrogate marker p16^INK4A^ was performed as reported by our group previously [15].

### DNA isolation and HPV typing by PCR

DNA isolation was performed using the Gentra puregene tissue kit with proteinase K treatment (Qiagen, Hilden, Germany) according to the manufacturer's instructions. The DNA was extracted from the paraffin-embedded samples or taken from fresh frozen samples if existent. Details were reported by our group previously [25].

### VCP Expression by qPCR

RNA isolated from 15 fresh frozen tumor samples where data from CGH were available [23] was reverse transcribed using the iScript cDNA Synthesis Kit (BioRad, Munich, Germany). qPCR reactions were performed using iTaq Universal SYBR Green Supermix (BioRad). The following VCP-specific primers were used: Forward primer 5′-AAACCGTGGTAGAGGTGCCA-3′ and reverse 5′-CTTGGAAGGTGTCATGCCAA-3′ [20]. The detection of the housekeeping gene β-Actin was used for normalization of mRNA levels: Forward primer 5′-GGACTTCGAGCAAGAGATGG-3′ and reverse 5′-CAGTGATCTCCTTCTGCATC-3′ [26].

### Statistical analysis

VCP, p16 and HPV-status were analyzed using cross-tabulations, chi-square test and Fisher's exact probability test with the software SPSS 21.0. Overall survival and disease-free survival rates were estimated using the Kaplan–Meier algorithm for incomplete observations. The overall survival time was measured from the date of diagnosis to the last date when the patient was known to be alive (censored) or date of death for any reason (uncensored). The disease-free survival rate was defined as the period of time beginning on the date of diagnosis to the day of the last follow-up examination in which the patient was disease-free (censored), or to the date of local recurrence of the disease or occurrence of regional or distant metastases (uncensored). The log-rank test was used to perform the univariate analysis of the various variables. A Cox proportional hazards ratio model was used to determine independent predictors of overall survival using factors significant on univariate analysis as covariates. To correct for multiple testing, p-values were adjusted using the Bonferroni correction. A corrected p-value of <0.05 was considered to be significant for all analysis.

## Results

### HPV Status

In 93 OSCC cases DNA integrity was sufficient. HPV were detected in 26.9% (n = 25) of 93 OSCC samples, 73.1% (n = 68) of 93 tissue samples showed a HPV-negative result (Table 1). These included HPV16 in 24 (97%) tissue samples and HPV33 in one tissue sample.

**Table 1:** Clinicopathological characteristics of the OSCC patients.

Clinicopathological characteristics	No. of Patients	%	N (Total)
**Sex**			106
Male	75	70.8	
Female	31	29.2	
**Age**			102
Median	57		
Range	34–79		
= <57	51	50.0	
>57	51	50.0	
**T-stage**			104
1	29	27.9	
2	29	27.9	
3	16	15.4	
4	30	28.8	
**N-stage**			101
0	16	15.8	
1	21	20.8	
2	49	48.5	
3	15	14.9	
**M-stage**			103
0	82	79.6	
1	7	6.8	
X	14	13.6	
**Treatment**			102
Surgery + RT/RCT	61	59.8	
RT/RCT alone	21	20.6	
Surgery alone	16	15.7	
No therapy	4	3.9	
**Regular smoking**			83
+	73	88.0	
−	10	12.0	
**Regular drinking**			83
+	72	86.7	
−	11	13.3	
**Absolute VCP- staining intensity**			106
0–1	50	47.2	
2–3	56	52.8	
**HPV detection and p16 overexpression**			93
−	68	73.1	
+	25	26.9	

### Treatment and clinical details of the patients

93 patients were treated in curative intention. 16 patients (15.7%) underwent a surgical therapy without adjuvant radio-/radiochemotherapy. 61 patients (59.8%) were treated by surgical and postoperative radio-/radiochemotherapy. In 21 cases (20.6%) a primary radiochemotherapy was used. Four patients (3.9%) did not undergo a specific therapy. More clinical details are given in Table 1.

### Survival outcome

The mean period of follow-up time was 34.2 months (0.3–131.5 months). The 5-years overall survival and disease-free survival rates were 54.6% and 71.2%, respectively.

### VCP-expression in OSCC

In total 106 samples were scrutinized carefully. We found a negative or weak VCP-expression in 47.2% (n = 50, level 0–1 staining). Level 2–3 staining was determined in 52.8% (n = 56) of all cases (Table 1). In most OSCC, we found a homogenous staining pattern throughout the entire specimen. Nontumourous oropharyngeal tissue constantly showed no VCP-expression.

### Correlation of VCP-expression level, HPV-status and TNM stage

We could not show a correlation of the absolute intensity of VCP-expression level (weak and high) with the stage of all tumors further analyzed to the pTNM classification (Table 2). Furthermore, no correlation could be found between high VCP-expression levels and the T-status, neither in the HPV-positive, nor in the HPV-negative nor in all OSCC samples.

**Table 2:** Absolute Valosin-contained protein (VCP) staining levels and TNM-stage/HPV-status in OSCC patients.

Patient characteristics	Total No. of Patients	Patients with absolute VCP level 0–1 expression	Patients with absolute VCP level 2–3 expression	p-value
**T-stage, n (%)**				
1	29	11 (37.9)	18 (62.1)	NS
2	29	11 (37.9)	18 (62.1)	
3	15	9 (60.0)	6 (40.0)	
4	29	17 (58.6)	12 (41.4)	
**N-status, n (%)**				
negative	16	7 (43.8)	9 (56.3)	NS
positive	83	38 (45.8)	45 (54.2)	
**M-stage, n (%)**				
0	80	36 (45.0)	44 (55.0)	NS
1	7	2 (28.6)	5 (71.4)	
**HPV detection and p16 overexpression, n (%)**				
−	66	29 (43.9)	37 (56.1)	NS
+	25	10 (40.0)	15 (60.0)	

A Bonferroni corrected p-value of 0.0125 was considered to be significant.

In addition no correlation between VCP-expression level and the HPV status could be shown (Table 2).

### Correlation of VCP-expression level, HPV-status and clinical data

**Figure 2:**
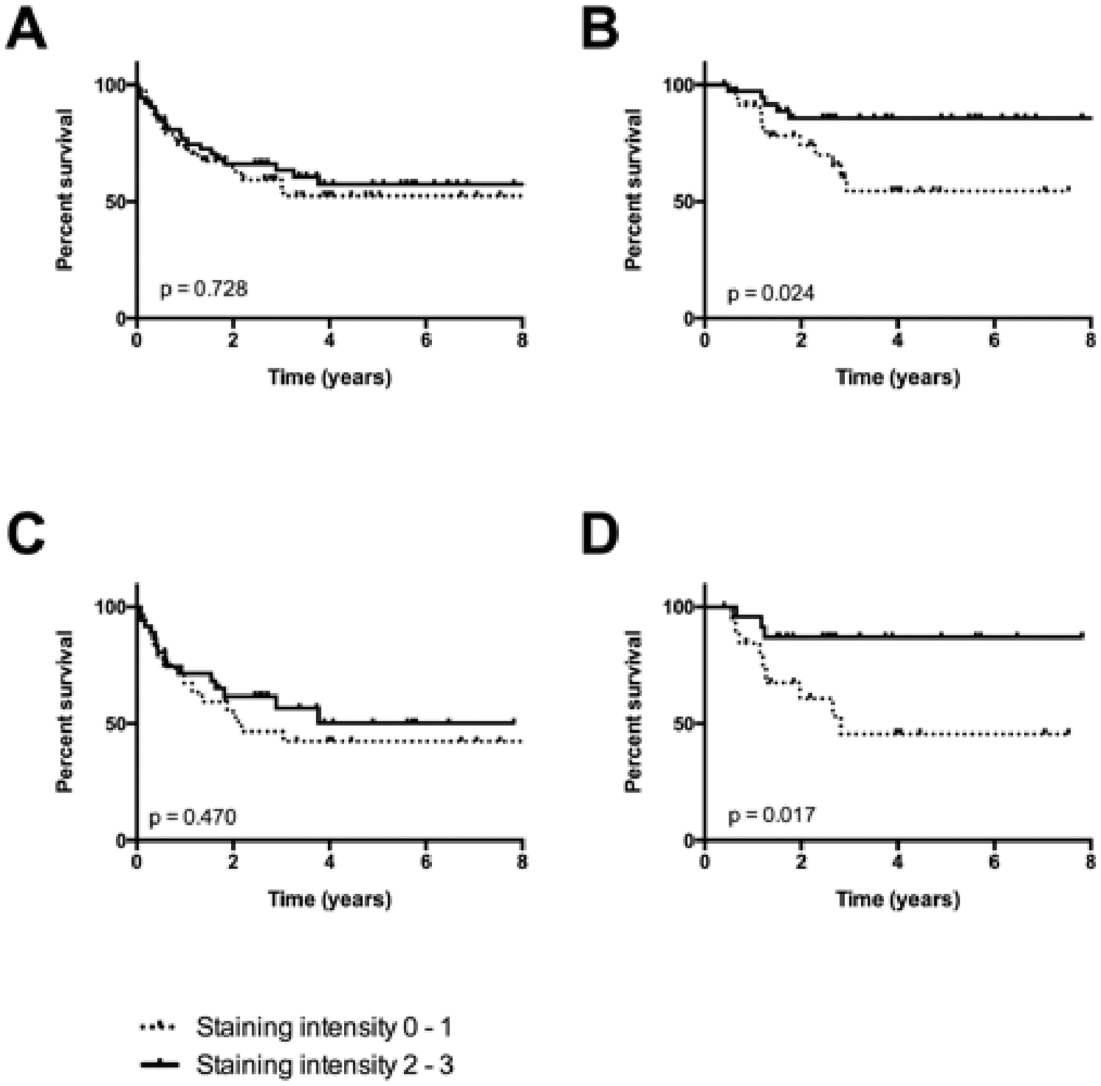
A: Overall survival of OSCC patients with absolute Valosin-containing protein (VCP)-expression level 0–1 and 2–3. No significance could be shown between the two groups (p = 0.728). **B:** Disease-free survival of OSCC patients with absolute Valosin-containing protein (VCP)-expression levels 0–1 and 2–3. The difference between the two groups is significant (p = 0.024). **C:** Overall survival of HPV-negative OSCC patients with absolute Valosin-containing protein (VCP)-expression level 0–1 and 2–3. No significance could be shown between the two groups (p = 0.470). **D:** Disease-free survival of HPV-negative OSCC patients with absolute Valosin-containing protein (VCP)-expression levels 0–1 and 2–3. The difference between the two groups is significant (p = 0.017). In all OSCC and in HPV-negative OSCC a Bonferroni corrected p-value of 0.025 was considered to be significant.

In 106 tissue samples we found a significant difference between patients with level 0–1 staining and level 2–3 staining expression regarding the disease-free survival (Figure 2A). Patients showing a low VCP-expression (level 1) seem to have a significantly poorer disease-free survival rate (p = 0.024) (Figure 2A). However a significant difference in overall survival between patients with VCP-expression level 0–1 and 2–3 could not be shown (p = 0.728, figure 2B).

In the sub-analysis of HPV-positive (26.9%) and HPV-negative OSCC (73.1%) it could be observed that there is a correlation between weak VCP intensity in HPV-negative patients and a significantly lower 5-year disease-free survival (45.6% vs. 86.4%, p = 0.017, figure 2C), whereas no significance could be demonstrated between HPV-positive OSCC and the staining level of VCP (100% vs. 82.50%, p = 0.260).

### VCP Expression by qPCR

**Figure 3:**
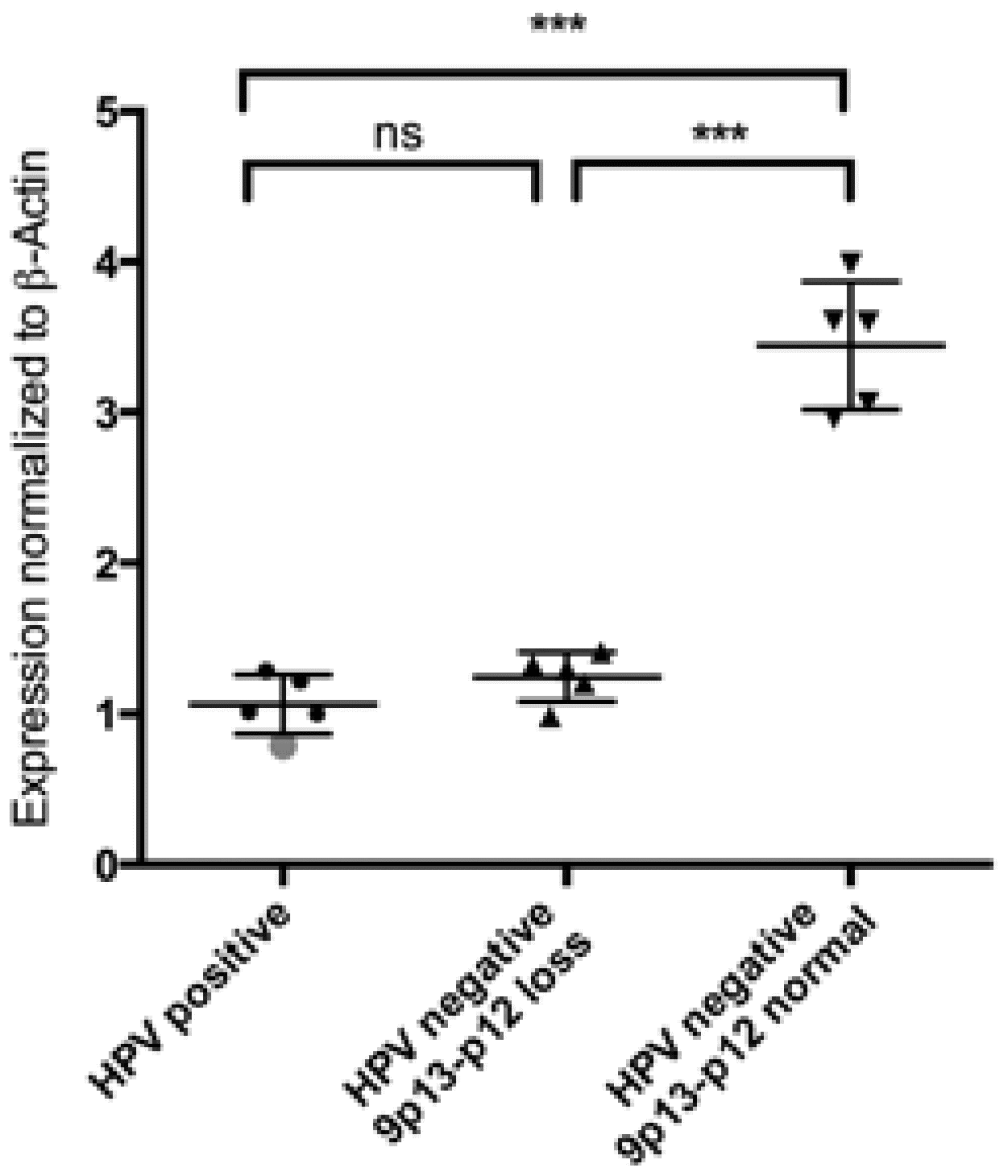
Expression of the VCP gene. qPCR has been performed on HPV-negative samples with known status of the 9p13-p12 region containing the VCP gene (5 samples each). Grey dot: HPV-positive sample with loss of 9p13-p12. HPV16-positive samples with no aberration of this locus were included for comparison (n = 5). Expression was normalized to β-Actin. *** p<0.001, Kruskal-Wallis test.

HPV-negative samples with a normal 9p13-p12 region showed a significantly higher VCP Expression normalized to β-Actin in comparison to HPV-negative samples with a loss of 9p13-p12 region (p<0.001, figure 3). HPV16-positive samples with no aberration of this locus were included for comparison (n = 5) and showed significantly lower VCP Expression (p<0.001, figure 3).

## Discussion

Oropharyngeal squamous cell carcinomas (OSCC) show great variety in terms of etiology, biological behavior and prognosis [27]. At least one third of OSCC are HPV-induced cancers, predominantly caused by high-risk HPV type 16 [3]–[5], [28]–[31]. Recent studies estimate that the prognosis is superior in HPV-positive OSCC compared to HPV-negative OSCC [32]. A reason for this may be the better response to radiotherapy and chemotherapy, even though HPV-positive patients present with a more advanced stage of disease [33]–[35]. The tumor-node-metastasis (TNM) staging system is a worldwide accepted classification scheme and is used as standard method to predict prognosis [4]. However prognoses of patients are inconsistent even if they are classified in the same TNM-stage. Therefore several clinical and molecular factors were reported in malignancies to reinforce the TNM staging system. For oropharyngeal malignancies recent studies evaluated survivin expression, podoplanin expression and other pivotal cell-cycle proteins regarding the predictive potential on prognosis [12]–[15]. VCP-Expression was described as a suitable marker for prediction of grade of metastasis and prognosis in esophageal, gastric, prostate and lung carcinoma [18]–[21].

Yamamoto et al. reported that gastric carcinoma with a higher expression of VCP showed higher rates of lymph node metastasis, deep tumor invasion and poorer overall survival and disease-free survival compared with gastric carcinoma with lower expression of VCP [19]. In 2004, Yamamoto et al. could show a higher rate of lymph node metastasis, deep tumor invasion, poorer disease-free survival and overall survival in high VCP expression level esophageal squamous cell carcinoma (ESCC) compared to low VCP expression level ESCC [18]. Our results show that a VCP overexpression in HPV-negative patients was associated with significantly better 5-year disease-free survival probability (86.4% vs. 45.6%, p = 0.017; figure 2). In contrast, patients with HPV-positive OSCC showed favorable prognosis regardless of VCP-expression level. These findings were unexpected and differ from previous reports of VCP-expression in esophageal, gastric, prostate and lung carcinoma [18]–[21] in which a higher VCP-expression level was correlated with a lower 5-year disease-free survival. Nevertheless, a study by our group showed that 61% of HPV-negative OSCC harboured a (partial) loss of chromosome 9p including 42% of cases with loss of the 9p13-p12 region amongst others harbouring the VCP gene. Whereas loss of 9p in general was correlated with better prognosis in HPV-positive OSCC and was not significantly correlated with prognosis in HPV-negative OSCC [23], re-evaluation of these data for 9p13-p12 loss revealed a worse prognosis in HPV-negative OSCC (data not shown). Therefore it is possible that there is a direct correlation between 9p13-p12 loss, weak VCP mRNA and protein expression and unfavourable survival in HPV-negative OSCC.

VCP effects a regulation of activation of NF
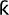
B. NF
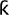
B is a transcription factor whose activity is involved in apoptosis, cell-proliferation and invasion [36]. Therefore Yamamoto et al. discuss that high levels of VCP may lead to high expression levels of NF
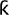
B. Thus, NF
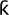
B-regulated gene products may lead to oral cancer metastasis [37]. VCP is also correlated with the ubiquitin/proteasome-dependent protein degradation pathway. Via intracellular pathways VCP is involved in regulation of the cell cycle progression, cell transformation and signal transduction [37].

The present study shows that HPV-positive OSCC show a high VCP-expression level (level 2–3) more often (60%) than weak expression (level 0–1; 40%) (Table 2). However, in HPV negative OSCC high VCP-expression level was determined in 56.1% and a weak VCP-expression level in 43.9% (Table 2).

Although lymph node metastasis are more often seen in VCP-expression level 2–3 (Table 2), a significant correlation between high VCP-expression rate in HPV-positive and HPV-negative OSCC and lymph node metastasis could not be shown in our study. Yamamoto et al. could demonstrate significantly higher frequencies of lymph node metastasis in higher VCP-expression level in esophageal squamous cell carcinoma versus weak VCP-expression level [18]. Furthermore they analyzed the intensity of immunochemistry staining of the specimens by comparison to non-carcinoma endothelial cells and categorized as follows: weaker (VCP-expression level 1) or equal to or stronger (level 2) than expression in non-carcinoma endothelial cells [18]–[21]. A possible explanation could be that a high level of VCP might lead to a degradation of inhibitor 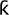
Bα (I
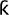
Bα). Hereby higher levels of NF
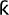
B are reached [16], [17]. NF
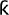
B modulates the factors IL-6, IL-8, GRO-1, VEGF and HGF in baseline serum samples [38]. A higher concentration of NF
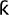
B elevates the baseline serum VEGF [38]. In addition VEGF may be increased by higher NF
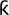
B and proinflammatory cytokines in epithelial cells by exposure to carcinogens in tobacco products [39], [40]. Interestingly, smoking is one of the most important risk factors for HPV-negative OSCC [4]–[6]. Allan et al. could show that elevation of baseline serum VEGF correlates with superior survival in advanced OSCC in patients treated by chemoradiation [38].

In conclusion, our study shows that VCP-expression level determined by immunohistochemistry seems to be a prognostic marker in HPV negative OSCC. Further studies are needed to gain insight into the molecular mechanisms that contribute to these findings to transfer them into clinical routine. The results of our study must however be interpreted carefully given the small sample size of our study subgroups.

## References

[1] Ferlay J, Shin HR, Bray F, Forman D, Mathers C, et al (2010) Estimates of worldwide burden of cancer in 2008: GLOBOCAN 2008. Int J Cancer 127:2893–2917. 21351269 10.1002/ijc.25516

[2] Shiboski CH, Schmidt BL, Jordan RC (2005) Tongue and tonsil carcinoma: increasing trends in the U.S. population ages 20–44 years. Cancer 103:1843–1849. 15772957 10.1002/cncr.20998

[3] Blomberg M, Nielsen A, Munk C, Kjaer SK (2011) Trends in head and neck cancer incidence in Denmark, 1978–2007: focus on human papillomavirus associated sites. Int J Cancer 129:733–741. 20878955 10.1002/ijc.25699

[4] Preuss SF, Klussmann JP, Semrau R, Huebbers C (2011) Update of HPV-induced oropharyngeal cancer. HNO 59:1031–1037. 21956679 10.1007/s00106-011-2391-z

[5] Smith EM, Ritchie JM, Summersgill KF, Klussmann JP, Lee JH, et al (2004) Age, sexual behavior and human papillomavirus infection in oral cavity and oropharyngeal cancers. Int J Cancer 108:766–772. 14696105 10.1002/ijc.11633

[6] D'Souza G, Kreimer AR, Viscidi R, Pawlita M, Fakhry C, et al (2007) Case-control study of human papillomavirus and oropharyngeal cancer. N Engl J Med 356:1944–1956. 17494927 10.1056/NEJMoa065497

[7] Klussmann JP, Dinh S, Guntinas-Lichius O, Wittekindt C, Weissenborn S, et al (2004) HPV-associated tonsillar cancer. An update. HNO 52:208–218. 15004685 10.1007/s00106-004-1069-1

[8] Hafkamp HC, Speel EJ, Haesevoets A, Bot FJ, Dinjens WN, et al (2003) A subset of head and neck squamous cell carcinomas exhibits integration of HPV 16/18 DNA and overexpression of p16INK4A and p53 in the absence of mutations in p53 exons 5–8. Int J Cancer 107:394–400. 14506739 10.1002/ijc.11389

[9] Gillison ML, Lowy DR (2004) A causal role for human papillomavirus in head and neck cancer. Lancet 363:1488–1489. 15135592 10.1016/S0140-6736(04)16194-1

[10] Bernier J, Domenge C, Ozsahin M, Matuszewska K, Lefèbvre JL, et al (2004) European Organization for Research and Treatment of Cancer Trial 22931. Postoperative irradiation with or without concomitant chemotherapy for locally advanced head and neck cancer. N Engl J Med 350:1945–1952. 15128894 10.1056/NEJMoa032641

[11] Eich HT, Löschcke M, Scheer M, Kocher M, Bongartz R, et al (2008) Neoadjuvant radiochemotherapy and radical resection for advanced squamous cell carcinoma of the oral cavity. Outcome of 134 patients. Strahlenther Onkol 184:23–29. 18188519 10.1007/s00066-008-1725-6

[12] Huang CF, Sun ZJ, Zhao YF, Chen XM, Jia J, et al (2010) Increased expression of peroxiredoxin 6 and cyclophilin A in squamous cell carcinoma of the tongue. Oral Dis 17:328–334. 20796224 10.1111/j.1601-0825.2010.01730.x

[13] Kreppel M, Kreppel B, Drebber U, Wedemayer I, Rothamel D, et al (2012) Podoplanin expression in oral leukoplakia: prognostic value and clinicopathological implications. Oral Dis 18:692–399. 22471854 10.1111/j.1601-0825.2012.01927.x

[14] Mashhadiabbas F, Mahjour F, Mahjour SB, Fereidooni F, Hosseini FS (2012) The immunohistochemical characterization of MMP-2, MMP-10, TIMP-1, TIMP-2, and podoplanin in oral squamous cell carcinoma. Oral Surg Oral Med Oral Pathol Oral Radiol 114:240–250. 22769410 10.1016/j.oooo.2012.04.009

[15] Preuss SF, Weinell A, Molitor M, Semrau R, Stenner M, et al (2008) Survivin and epidermal growth factor receptor expression in surgically treated oropharyngeal squamous cell carcinoma. Head Neck 30:1318–1324. 18704972 10.1002/hed.20876

[16] Asai T, Tomita Y, Nakatsuka S, Hoshida Y, Myoui A, et al (2002) VCP (p97) regulates NFkappaB signaling pathway, which is important for metastasis of osteosarcoma cell line. Jpn J Cancer Res 93:296–304. 11927012 10.1111/j.1349-7006.2002.tb02172.x PMC5926968

[17] Dai RM, Chen E, Longo DL, Gorbea CM, Li CC (1998) Involvement of valosin-containing protein, an ATPase co-purified with IkappaBalpha and 26 S proteasome, in ubiquitin-proteasome-mediated degradation of IkappaBalpha. J Biol Chem 273:3562–3573. 9452483 10.1074/jbc.273.6.3562

[18] Yamamoto S, Tomita Y, Hoshida Y, Iizuka N, Kidogami S, et al (2004) Expression Level of Valosin-Containing Protein (p97) Is Associated with Prognosis of Esophageal Carcinoma. Clin cancer Res 10:5558–5565. 15328197 10.1158/1078-0432.CCR-0723-03

[19] Yamamoto S, Tomita Y, Hoshida Y, Iizuka N, Monden M, et al (2004) Expression level of valosin-containing protein (p97) is correlated with progression and prognosis of non-small-cell lung carcinoma. Ann Surg Oncol 11:697–704. 15231524 10.1245/ASO.2004.10.018

[20] Yamamoto S, Tomita Y, Hoshida Y, Takiguchi S, Fujiwara Y, et al (2003) Expression level of valosin-containing protein is strongly associated with progression and prognosis of gastric carcinoma. J Clin Oncol 21:2537–2544. 12829673 10.1200/JCO.2003.12.102

[21] Tsujimoto Y, Tomita Y, Hoshida Y, Kono T, Oka T, et al (2004) Elevated expression of Valosin-containing protein (p97) is associated with poor prognosis of prostate cancer. Clin Cancer Res 10:3007–3012. 15131036 10.1158/1078-0432.ccr-03-0191

[22] Jing M, Bohl J, Brimer N, Kinter M, Vande Pol SB (2007) Degradation of tyrosine phosphatase PTPN3 (PTPH1) by association with oncogenic human papillomavirus E6 proteins. J Virol 81:2231–2239. 17166906 10.1128/JVI.01979-06 PMC1865939

[23] Klussmann JP, Mooren JJ, Lehnen M, Claessen SM, Stenner M, et al (2009) Genetic signatures of HPV-related and unrelated oropharyngeal carcinoma and their prognostic implications. Clin Cancer Res 15:1779–86. 19223504 10.1158/1078-0432.CCR-08-1463

[24] American Joint Commitee on Cancer. Cancer Staging Manual- Seventh Edition of OSCC [Internet]. Available: http://www.cancerstaging.org; http://web2.facs.org/cstage0205/oropharynx/Oropharynx_upt.html Accessed 2013 Oct 01.

[25] Meyer MF, Huebbers CU, Siefer OG, Vent J, Engbert I, et al (2014) Prevalence and risk factors for oral human papillomavirus infection in 129 women screened for cervical HPV infection. Oral Oncol 50:27–31. 24169586 10.1016/j.oraloncology.2013.10.009

[26] Häfner N, Driesch C, Gajda M, Jansen L, Kirchmayr R, et al (2008) Integration of the HPV16 genome does not invariably result in high levels of viral oncogenetranscripts. Oncogene 27:1610–1617. 17828299 10.1038/sj.onc.1210791

[27] Forastiere A, Koch W, Trotti A, Sidransky D (2001) Head and neck cancer. N Engl J Med 345:1890–1900. 11756581 10.1056/NEJMra001375

[28] Kreimer AR, Clifford GM, Boyle P, Franceschi S (2005) Human papillomavirus types in head and neck squamous cell carcinomas worldwide: a systematic review. Cancer Epidemiol Biomarkers Prev 14:467–475. 15734974 10.1158/1055-9965.EPI-04-0551

[29] Mellin H, Dahlgren L, Munck-Wikland E, Lindholm J, Rabbani H, et al (2002) Human papillomavirus type 16 is episomal and a high viral load may be correlated to better prognosis in tonsillar cancer. Int J Cancer 102:152–158. 12385011 10.1002/ijc.10669

[30] Gillison ML, Koch WM, Capone RB, Spafford M, Westra WH, et al (2000) Evidence for a causal association between human papillomavirus and a subset of head and neck cancers. J Natl Cancer Inst 92:709–720. 10793107 10.1093/jnci/92.9.709

[31] Li W, Thompson CH, Cossart YE, O'Brien CJ, McNeil EB, et al (2004) The expression of key cell cycle markers and presence of human papillomavirus in squamous cell carcinoma of the tonsil. Head Neck 26:1–9. 14724900 10.1002/hed.10335

[32] Lindel K, Beer KT, Laissue J, Greiner RH, Aebersold DM (2001) Human papillomavirus positive squamous cell carcinoma of the oropharynx: a radiosensitive subgroup of head and neck carcinoma. Cancer 92:805–813. 11550151 10.1002/1097-0142(20010815)92:4<805::aid-cncr1386>3.0.co;2-9

[33] Klussmann JP, Weissenborn SJ, Wieland U, Dries V, Kolligs J, et al (2001) Prevalence, Distribution, and Viral Load of Human Papillomavirus 16 DNA in Tonsillar Carcinomas. Cancer 92:2875–2884. 11753961 10.1002/1097-0142(20011201)92:11<2875::aid-cncr10130>3.0.co;2-7

[34] Chaturvedi AK, Engels EA, Pfeiffer RM, Hernandez BY, Xiao W, et al (2011) Human papillomavirus and rising oropharyngeal cancer incidence in the United States. J Clin Oncol 29:4294–4301. 21969503 10.1200/JCO.2011.36.4596 PMC3221528

[35] Ang KK, Harris J, Wheeler R, Weber R, Rosenthal DI, et al (2010) Human papillomavirus and survival of patients with oropharyngeal cancer. N Engl J Med 363:24–35. 20530316 10.1056/NEJMoa0912217 PMC2943767

[36] Mayo MW, Baldwin AS (2000) The transcription factor NF-kappaB: control of oncogenesis and cancer therapy resistance. Biochim Biophys Acta 1470:M55–M62. 10722927 10.1016/s0304-419x(00)00002-0

[37] Dai RM, Li CC (2001) Valosin-containing protein is a multi-ubiquitin chain-targeting factor required in ubiquitin-proteasome degradation. Nat Cell Biol 3:740–744. 11483959 10.1038/35087056

[38] Allen C, Duffy S, Teknos T, Islam M, Chen Z, et al (2007) Nuclear factor-kappaB-related serum factors as longitudinal biomarkers of response and survival in advanced oropharyngeal carcinoma. Clin Cancer Res 13:3182–3190. 17545521 10.1158/1078-0432.CCR-06-3047

[39] Anto RJ, Mukhopadhyay A, Shishodia S, Gairola CG, Aggarwal BB (2002) Cigarette smoke condensate activates nuclear transcription factor-nB through phosphorylation and degradation of InBa: correlation with induction of cyclooxygenase-2. Carcinogenesis 23:1511–1518. 12189195 10.1093/carcin/23.9.1511

[40] Yang SR, Chida AS, Bauter MR, Shafiq N, Seweryniak K, et al (2006) Cigarette smoke induces proinflammatory cytokine release by activation of NF-nB and posttranslational modifications of histone deacetylase in macrophages. Am J Physiol Lung Cell Mol Physiol 291:L46–57. 16473865 10.1152/ajplung.00241.2005

